# Continent‐wide patterns of song variation predicted by classical rules of biogeography

**DOI:** 10.1111/ele.14102

**Published:** 2022-09-20

**Authors:** Matteo Sebastianelli, Sifiso M. Lukhele, Emmanuel C. Nwankwo, Louis Hadjioannou, Alexander N. G. Kirschel

**Affiliations:** ^1^ Department of Biological Sciences University of Cyprus Nicosia Cyprus; ^2^ University of California Los Angeles Department of Ecology and Evolutionary Biology Los Angeles California USA; ^3^ Edward Grey Institute, Department of Zoology University of Oxford Oxford UK

**Keywords:** acoustic adaptation, Allen's rule, Bergmann's rule, bird song, body size, innate vocalisations, macroecology, peak frequency, thermoregulation, tinkerbirds

## Abstract

Physiological constraints related to atmospheric temperature pose a limit to body and appendage size in endothermic animals. This relationship has been summarised by two classical principles of biogeography: Bergmann's and Allen's rules. Body size may also constrain other phenotypic traits important in ecology, evolution and behaviour, and such effects have seldom been investigated at a continental scale. Through a multilevel‐modelling approach, we demonstrate that continent‐wide morphology of related African barbets follows predictions of Bergmann's rule, and that body size mirrors variation in song pitch, an acoustic trait important in species recognition and sexual selection. Specifically, effects on song frequency in accordance with Bergmann's rule dwarf those of acoustic adaptation at a continental scale. Our findings suggest that macroecological patterns of body size can influence phenotypic traits important in ecology and evolution, and provide a baseline for further studies on the effects of environmental change on bird song.

## Graphical abstract

Temperature‐related physiological constraints shape functional traits in animals at a global scale, yet the implications of continent‐wide morphological variation on acoustic communication signals is little understood. This is despite a well‐established relationship between body size and acoustic signal frequencies across the animal kingdom. In a continent‐wide study on birds, we show that song frequency mirrors predictable patterns of body size variation along a latitudinal and elevational gradient, suggesting climate change may drive predictable shifts in the frequency of acoustic signals that play a critical role in animal ecology and evolution.

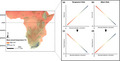

## INTRODUCTION

Diverse organisms respond analogously to environmental pressures at a global scale, providing evidence that geographical variation in phenotypes arises from natural selection (Guillaumet et al., 2005; Kelly, 2006; Novembre & Di Rienzo, 2009). Thermal homeostasis, which is the ability to maintain body temperature within a narrow range regardless of ambient temperature, represents a major constraint in endothermic animals that is critical for survival (McKechnie & Wolf, 2010). This is because an organism's activity is constrained by the sustainability of metabolic processes as well as its capacity to exchange heat with the atmosphere (Speakman & Król, 2010), with the latter process tightly linked with morphology (Mitchell et al., 2018).

The relationship between morphology and the physiological need to maintain constant temperature becomes more evident when scrutinising macroecological patterns of variation in body and appendage size. Two major ecogeographical rules have been formulated to describe such trends of morphological variation at a global scale: Bergmann's (Bergmann, 1847) and Allen's rules (Allen, 1877). Bergmann's rule posits that larger body sizes are found at higher latitudes and elevation, because larger bodies facilitate retention of body heat in cooler climates with their smaller body surface area to body volume ratio. Based on similar principles, Allen's rule states that larger appendages (i.e. beaks, tails and unfurred limbs) relative to body size help exchange body heat with the atmosphere and therefore relatively larger appendages are more advantageous at lower latitudes (and elevation), where temperatures are higher, than towards the poles. Both ecogeographic rules have been supported by studies on birds (Ashton, 2002; Nudds & Oswald, 2007; Olson et al., 2009; Romano et al., 2020, 2021; Tobias et al., 2022) and mammals (Alhajeri et al., 2020; Meiri & Dayan, 2003), as well as other taxa (Alho et al., 2011; Osorio‐Canadas et al., 2016). However, many exceptions have also been reported (Ashton & Feldman, 2003; Freeman, 2017; Gutiérrez‐Pinto et al., 2014; Nunes et al., 2017), possibly because of complex ecological and evolutionary trade‐offs between body and limb size, and resource availability, predation risk, dispersal ability, resistance to starvation or even environmental factors that affect species richness (Greve et al., 2008; Nunes et al., 2017; Romano et al., 2021). Nonetheless, a growing number of studies have focused on patterns of widescale spatial variation in morphological traits because it allows researchers to predict organismal responses to a constantly changing climate (Weeks et al., 2020), which could result from changes in developmental stage (Møller et al., 2018), and represent either non‐adaptive (Teplitsky & Millien, 2014) or adaptive responses (Bay et al., 2018; Jirinec et al., 2021; Price et al., 1984).

Although these ecogeographic rules have been widely tested both within (Romano et al., 2020, 2021) and across species of terrestrial animals (Alhajeri et al., 2020; Ashton, 2002), with Bergmann's rule tested even on ectotherms (Berke et al., 2013; Campbell et al., 2021), it remains unclear whether macroecological temperature‐dependent variation in morphology might drive continental‐scale variation in behavioural traits.

Physiologically constrained variation in body size likely affects animal ecology and therefore its evolution. But it might also influence other selected traits through correlational selection, which occurs when the effect of a specific trait on individual fitness depends on its interaction with a second trait (McGlothlin et al., 2005). Bird song is a labile behavioural trait that mediates key interactions such as mate attraction (Catchpole & Slater, 1995) and therefore assortative mating (Slabbekoorn & Smith, 2002a), which contribute to the maintenance of reproductive isolation between species (Janicke et al., 2019). By consequence, any mechanism that leads to variation in acoustic traits may have implications for speciation (West‐Eberhard, 1983).

Morphology is strongly correlated with some acoustic traits. Specifically, there is considerable evidence supporting a negative relationship between body size and song pitch, as well as in beak size with both pitch (Friedman et al., 2019; Kirschel, Zanti, Harlow, et al., 2020; Mikula et al., 2021; Ryan & Brenowitz, 1985; Uribarri et al., 2020) and song pace (Derryberry et al., 2012; Podos, 2001). This is because body size is associated with the size of the syringeal membrane and by consequence to the fundamental frequency of song (Ryan & Brenowitz, 1985). Similarly, due to biomechanical constraints, larger beaks are associated with slower‐paced songs (Derryberry et al., 2012; Podos, 2001), but are also associated with lower frequencies, because the suprasyringeal tract which includes the trachea, glottis and buccal cavity, has resonating properties that affect song frequency (Nowicki, 1987; Palacios & Tubaro, 2000). Besides intrinsic effects, bird song has also been shown to vary because of numerous extrinsic mechanisms. For instance there is much evidence of background noise shaping acoustic signals, including from both biotic and abiotic natural sounds (Kirschel et al., 2011; Kirschel, Blumstein, Cohen, et al., 2009; Sebastianelli et al., 2021; Slabbekoorn & Smith, 2002b; Smith et al., 2013), and anthropogenic noise (Nemeth & Brumm, 2009; Slabbekoorn & Peet, 2003). Likewise, interactions between closely related species may drive divergent character displacement in vocal signals facilitating species recognition (Kirschel, Blumstein, & Smith, 2009) or even convergent character displacement facilitating competitor recognition (Kirschel et al., 2019). Moreover, acoustic adaptation to sound transmission properties of the environment is another well‐tested hypothesis, albeit with mixed support. Densely vegetated habitats are associated with attenuation of higher frequencies and greater reverberation of sounds (Morton, 1975; Slabbekoorn et al., 2002; Wiley & Richards, 1982) and therefore, as summarised in the acoustic adaptation hypothesis (AAH), acoustic signals in dense habitats are predicted to have lower frequencies, slower pace and simpler structure compared to sounds produced in less densely vegetated habitats (Kirschel, Blumstein, Cohen, et al., 2009; Mikula et al., 2021; Seddon, 2005; Tobias et al., 2010). However, a recent analysis on more than 5000 passerine species found a weak positive association between habitat type and peak song frequency in passerines (Mikula et al., 2021), in contrast to AAH predictions. Lastly, although body mass, background noise and the sensory drive hypothesis might predict song patterns across space, such trends might be offset by the ability to learn songs from conspecific individuals by means of auditory feedback—that is, vocal learning (Jarvis, 2004; Nowicki & Searcy, 2014). Indeed, vocal learning can lead to the development and fixation of new dialects in relatively short periods of time (Logue & Leca, 2020; Mennill et al., 2018), an ability attributed to oscine passerines, parrots and hummingbirds (Catchpole & Slater, 1995; Mennill et al., 2018).

**FIGURE 1:**
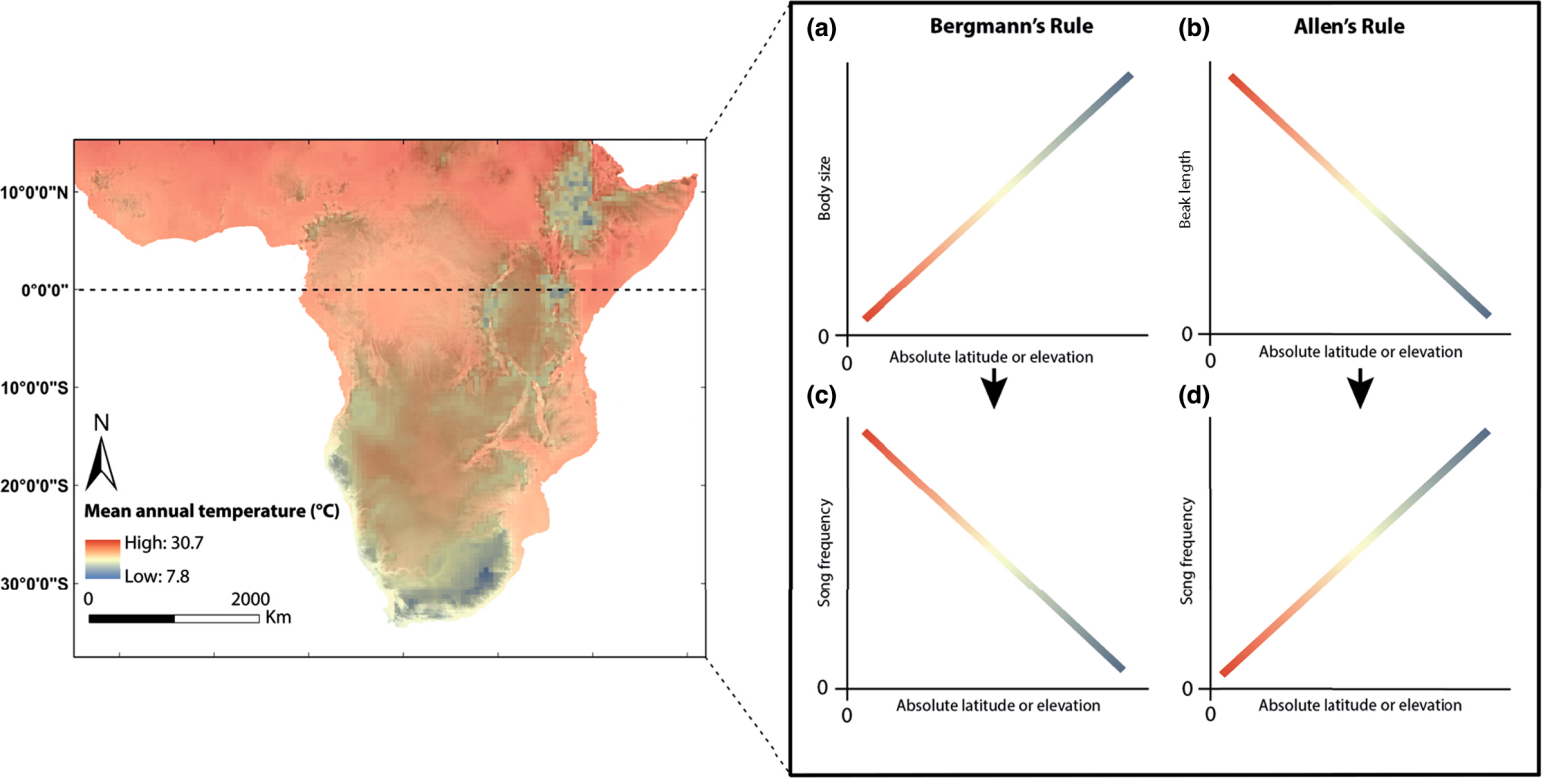
Mean annual temperature map of sub‐Saharan Africa from GHCN‐CAMS (Fan & van den Dool, 2008) illustrating how temperature decreases with increasing latitude and elevation. Two possible mechanisms can lead to predictable variation of song frequency across space following Bergmann's or Allen's rules predictions. According to Bergmann's rule, increasing body size would result in lower frequency songs towards higher latitudes and elevation (a, c). By contrast, Allen's rule predicts that birds with longer beaks relative to body size, found at lower latitudes and elevation, sing lower frequency songs (b, d)

Although there is some mixed support for the role of transmission properties in shaping acoustic signals (Ey & Fischer, 2009), and proximity to certain noise profiles might lead to predictable signal structure patterns (Kirschel et al., 2011; Smith et al., 2013), no study has examined whether temperature‐dependent variation in body size as predicted by broad biogeographical rules might underpin continent‐wide variation in acoustic signals. If such patterns emerged, they would suggest climate change may shape acoustic signals important in species recognition and mate choice. Here, we assess the extent to which continental‐scale variation in the body size, beak size and acoustic signals of four related species of *Pogoniulus* tinkerbirds, with distributions across a gradient of vegetation density throughout sub‐Saharan Africa (Short & Horne, 2001), and whose songs develop innately (Kirschel, Blumstein, & Smith, 2009), follow predictions of Bergmann's and Allen's rules (Figure 1). Using a mixed‐modelling approach, we investigated this question using data collected both from museum specimens and sound repositories, and during 17 years of fieldwork across sub‐Saharan Africa. We incorporated environmental remote sensing data in our models to account for potential effects of niche specialisation and acoustic adaptation.

## MATERIAL AND METHODS

### Morphometric data

**FIGURE 2:**
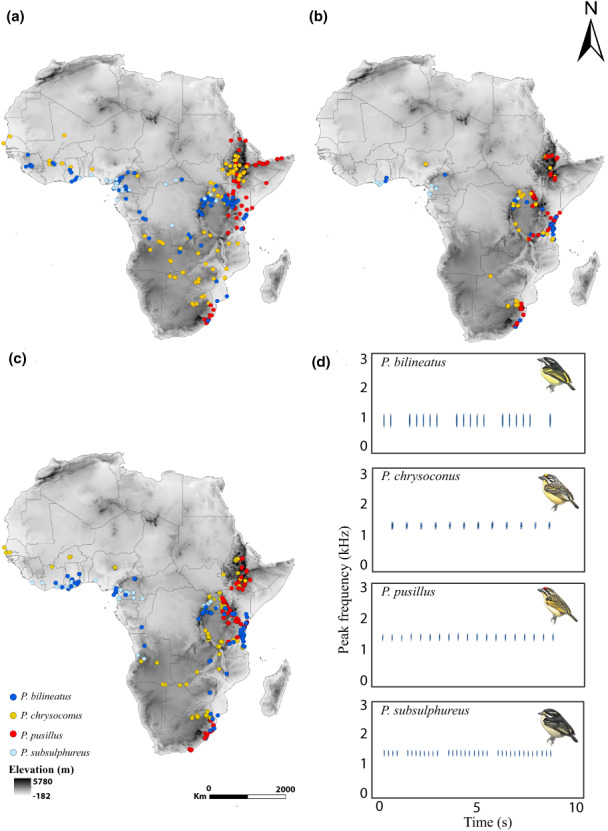
Map showing localities where tinkerbirds were sampled, including (a) the collection localities of museum specimens, (b) field collected samples, (c) recording localities, and (d) examples of song spectrograms for the four species, from Song ID: MW10_68_10 for *P. bilineatus*; 1007_44337 for *P. chrysoconus*; 1022_4439 for *P. pusillus* and AN05_009_04 for *P. subsulphureus*. The background map represents the digital elevation model (3‐arc second) of Africa. Tinkerbird illustrations courtesy of Lynx Edicions (del Hoyo et al., 2020)

We obtained continent‐wide morphometric data (Figure 2a) from 668 tinkerbird specimens (*P. bilineatus*: 329; *P. chrysoconus*: 138; *P. pusillus*: 117 and *P. subsulphureus*: 84) most of which were measured for previous studies (Kirschel et al., 2021; Kirschel, Nwankwo, Seal, & Grether, 2020; Nwankwo et al., 2018), from ornithology collections (Table S1, see also https://doi.org/10.6084/m9.figshare.20066225). In parallel with our morphometric measurements from museum study skins, we also took biometrics of 669 tinkerbirds of the four species that were ringed between 2004 and 2021 during extensive fieldwork across the African continent (*P. bilineatus*: 135; *P. chrysoconus*: 215; *P. pusillus*: 270 and *P. subsulphureus*: 49) (Kirschel, Blumstein, & Smith, 2009; Kirschel, Nwankwo, Seal, & Grether, 2020; Nwankwo et al., 2019) (Figure 2b). We pooled all data from *P. chrysoconus* and *P. pusillus* into a single taxon for our statistical analysis, for two reasons. First, the two species hybridise extensively in contact zones where many of our field measurements were obtained (Kirschel, Nwankwo, Pierce, et al., 2020; Nwankwo et al., 2019), meaning many individuals had mixed ancestry of varying proportions, and could not reliably be assigned to either species, and second, the monophyly of each currently recognised species in this group has recently been brought into question (Kirschel et al., 2021), rendering possible analyses at the species level spurious.

From museum specimens, we measured wing, tarsus, tail and beak length (exposed maxilla); from the field we took those same measurements and body mass. Although museum specimens were all measured by a single observer (ANGK), field biometrics were taken by five observers following standard protocols (Kirschel, Blumstein, & Smith, 2009; Nwankwo et al., 2019). As a result, some observer bias was inevitable, and we assessed potential observer bias in our measurements using a two‐step approach (see Supplemental Methods). The process resulted in removal of 14 outliers, with a final field biometrics dataset of 655 observations remaining.

### Acoustic data and song analysis

A total of 1588 tinkerbird songs were recorded (Figure 2c) between 2004 and 2021 (*P. bilineatus*: 379, *P. chrysoconus*: 388, *P. pusillus*: 536 and *P. subsulphureus*: 285, as identified in the field based on plumage) towards studies focusing on specific regions or species (Kirschel et al., 2021; Kirschel, Blumstein, & Smith, 2009; Kirschel, Nwankwo, Seal, & Grether, 2020; Nwankwo et al., 2018; Sebastianelli et al., 2021). We added 124 recordings from online repositories (e.g. http://www.xeno‐canto.org) and private collections (see recordings table: https://doi.org/10.6084/m9.figshare.20066225). We acknowledge that compiling recordings from different sources involves different observers, devices and file formats, thus raising the possibility of measurement error. However, a recent study has shown that sound compression (e.g. MP3 format) does not affect acoustic measurements such as peak frequency, though it may affect certain extreme values (Araya‐Salas et al., 2019). We avoided potential pitfalls in measurement bias by following recommendations therein. Furthermore, the use of different recording equipment might affect attenuation of high and low frequency but not peak frequency, with several recent studies analysing peak frequency differences at continental or global scales using recordings from an assortment of sources, recording equipment and file compression formats (Kirschel et al., 2019; Mikula et al., 2021).

Because *P. chrysoconus* and *P. pusillus* hybridise where they meet, species identification based on plumage might not reflect the genotype of individual birds, and their songs are also similar (see Figure 2d; Kirschel et al., 2021), even described as indistinguishable (Short & Horne, 2001, 2002). As for morphology, we thus treat *P. chrysoconus* and *P. pusillus* as one taxon in all our statistical models on song frequency. Field recordings of *P. bilineatus* and *P. subsulphureus* were measured previously using a protocol described therein for those species (Kirschel, Blumstein, & Smith, 2009; Nwankwo et al., 2018; Sebastianelli et al., 2021), while recordings attributed to *P. chrysoconus* and *P. pusillus*, as well as those from other sources for *P. bilineatus* and *P. subsulphureus*, were imported into Raven Pro 1.6 (Center for Conservation Bioacoustics, 2019) and individual notes detected using an automated energy detector (see Supplemental Methods for more details on recording methods and acoustic analysis).

### Environmental remote sensing data

Field localities were georeferenced with ±10 m accuracy. We then used the *extract values to points* function in ArcMap 10.7 (ESRI, 2011) to extract environmental remote sensing data. Because of spatial uncertainty with the museum dataset and recordings from online repositories and private collections, we interpolated extracted values from adjacent pixels to minimise overspecificity from those localities. From the Moderate‐Resolution Imaging Spectroradiometer (MODIS) we obtained enhanced vegetation index (EVI) at 250 m^2^ resolution, leaf area index (LAI, 500 m^2^ resolution) and percent tree cover (VCF, 250 m^2^ resolution). Elevation data were obtained at 30 m^2^ resolution (1‐arc second) from the global Shuttle Radar Topography Mission (SRTM) (Farr et al., 2007), and annual mean temperature (0.5° Latitude × 0.5° Longitude resolution) was provided by *NOAA/OAR/ESRL PSL, Boulder, Colorado, USA* (see Supplemental Methods for detailed descriptions of data and interpolation used).

### Statistical analysis

We tested our hypotheses using generalised linear mixed models (GLMMs) implemented in *glmmTMB* (Brooks et al., 2017) in R (v 4.1.3) (R Core Team, 2020). To test for variation in body size as predicted by Bergmann's rule, we first conducted principal component analyses (PCA) using the *prcomp* function in R. PCA was performed on body mass, tarsus, tail and wing length measurements separately on field and museum data (minus body mass and removal of three individuals with missing measurements from latter) after assessing the suitability of variables for PCA through Bartlett's sphericity test and estimating the Kaiser–Meyer–Olkin index. Principal components were selected based on eigenvalues >1 and used as dependent variables in Gaussian GLMMs as indicators of body size. This resulted in PC1 representing body size in all subsequent analyses. Taxon, absolute latitude, elevation and vegetation structure variables (EVI, LAI and VCF) were included as fixed factors, with individual nested in location as random factors to account for differences between individuals and among populations, and observer included in the model as either a fixed or crossed random effect to account for observer bias. We tested for effects of Allen's rule using beak length, since beaks are known to play an important role in bird thermoregulation (Ryding et al., 2021; Tattersall et al., 2009), and included the above fixed and random factors plus PC1 for body size, because Allen's rule predicts beak length should vary in relation to body size, included as a fixed factor. We note that museum specimen models excluded environment data, because many were collected before 1960 and thus prior to the earliest available MODIS data. These models excluded observer too, since one observer measured all specimens. Because variation in body size and appendage length attributed to Bergmann's and Allen's rules is linked to temperature, we replicated the above models replacing latitude and elevation with mean annual temperature, and also fitted a linear model to corroborate mean annual temperature was predicted by latitude and elevation.

We then tested for the effects of absolute latitude, elevation and the environment variables on peak song frequency across taxa, including interactions of each taxon with environmental and geographical variables to test for variation in the influence of AAH and ecogeographical effects on song among taxa, with individual nested in location as random factors. However, song frequency may vary between related species because of evolved differences in isolation (Edwards et al., 2005), or character displacement reducing interference in sympatry (Kirschel, Blumstein, & Smith, 2009). Such species‐level effects may obscure environmental effects in a multiple species model. Furthermore, respective species distributions could covary with ecological, elevational or latitudinal gradients, potentially obscuring environmental or ecogeographical effects. We thus also tested for geographical and environmental effects on song frequency for each taxon separately. In all song models, peak frequency was log‐transformed because its perception in birds is better represented on a logarithmic scale (Cardoso, 2013). For details on model selection, including full models, diagnostics, data visualisation and tests for effect sizes, see Supplemental Methods.

### Detecting phylogenetic signal

Phylogenetic relationships affect trait variation, with trait values of closely related species expected to be more similar than those of distantly related lineages. To account for this phylogenetic signal, we downloaded the phylogenetic tree (http://www.birdtree.org, Ericson backbone) that best reflected relationships among our study taxa based on previously published phylogenies (Kirschel et al., 2018, 2021; Kirschel, Nwankwo, Seal, & Grether, 2020; Nwankwo et al., 2019). We calculated Blomberg's *K* (Blomberg et al., 2003) for species specific values of peak frequency and field and museum morphometrics separately using the *picante* package in R (Kembel et al., 2010), setting number of randomisations to 1000. Values of *K* = 1 indicate that variation across the phylogenetic tree equals the one deriving from Brownian motion, whereas values of *K* < 1 or *K* > 1 indicate that phylogeny predicts less or more trait variation than expected under Brownian motion respectively.

## RESULTS

### Geographical variation in body and appendage size

**FIGURE 3:**
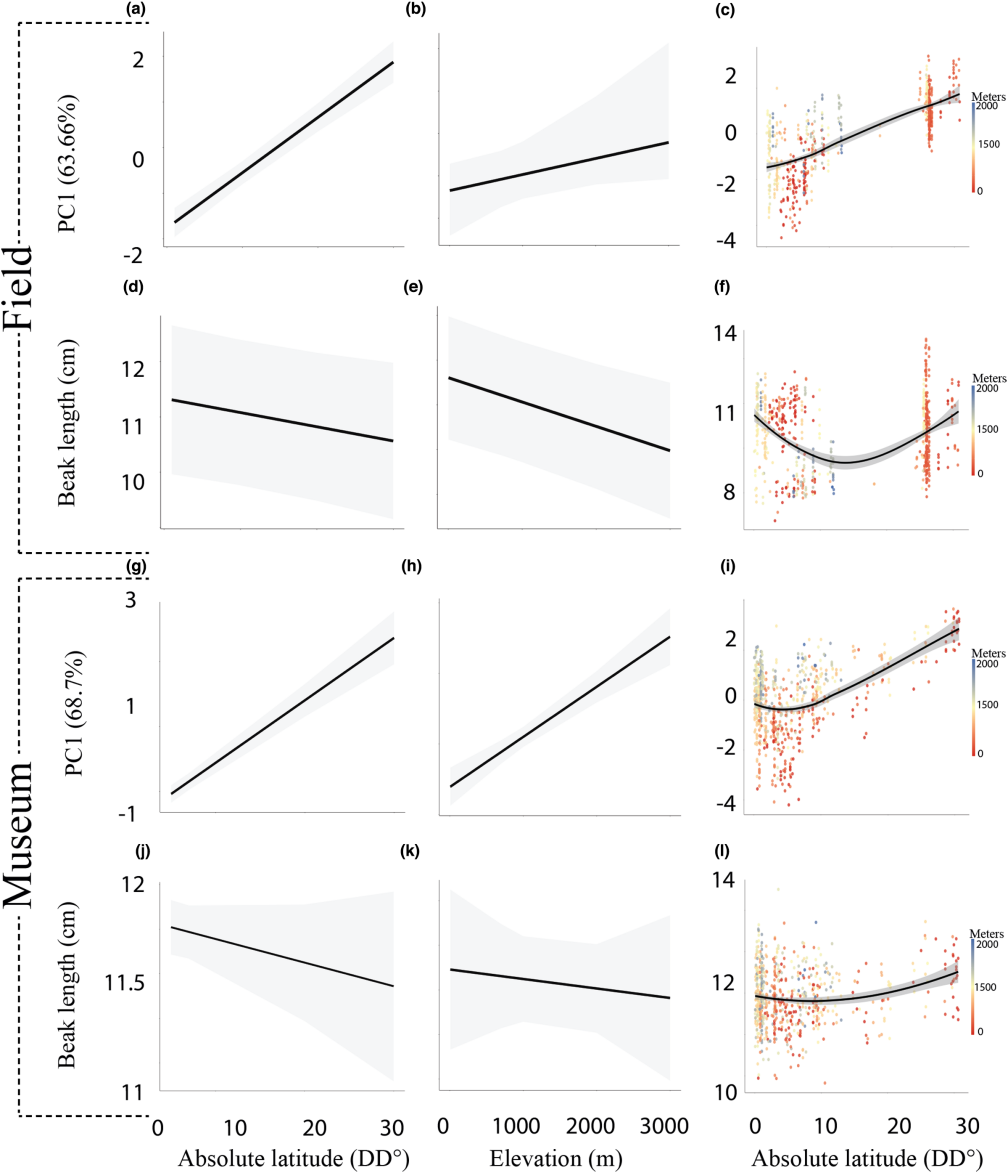
Plots showing the model effects of absolute latitude and elevation on body size as defined by PC1 in both field (a, b) and museum (g, h) measurements respectively as well as their respective effects on beak length after controlling for body size (d, e and j, k). Raw data plots with latitude are shown in the rightmost column (c, f, i, l), with elevation represented by the colour scale legend. Grey shaded area indicates 95% confidence interval

Variation in body size in all tinkerbird taxa closely followed predictions of Bergmann's rule (Figure 3a–c,g–i). PC1 extracted from PCA from field biometrics (positively associated with body mass and wing length, Table S3) was positively related with absolute latitude and elevation, while PC1 extracted from museum morphometrics (positively associated with tail and wing length, Table S3) was positively related with absolute latitude and elevation. Both results demonstrated that tinkerbirds are smaller towards the equator and at lower elevation, with a stronger effect of latitude (Table 1a,b). Strikingly, body size decreased from the southern hemisphere towards the equator and increased going north from there (Figure S1). We also found a significant difference among taxa, with *P. subsulphureus*, but not *P. chrysoconus* and *P. pusillus* combined, smaller than *P. bilineatus*.

**TABLE 1:** Best fit GLMM outputs showing the effects of absolute latitude, taxon and elevation on (a) body size PC1 from field collected data (which includes tarsus, tail, wing length and body mass), (b) body size PC1 from museum measurements (which includes tarsus, tail and wing length), (c) beak length from ringing data (with observer as random effect—for best fit model see Table S4) and (d) beak length from museum study skins. The relative importance of the predictors (i.e. Marginal *R* ^2^) and the conditional *R* ^2^ (Cond. *R* ^2^) are also reported

	Estimate	SE	*z*	*p*	Marginal *R* ^2^	Cond. *R* ^2^
**(a)**						
**Field body size (PC1: 63.66%)**					0.70	0.96
Intercept	−1.444	0.151	−9.525	<0.001		
Absolute latitude	0.112	0.007	14.068	<0.001	0.55	
Elevation	0.370	0.067	5.502	<0.001	0.001	
Taxon:					0.07	
*P. chrysoconus + P. pusillus*	−2.091	0.125	−0.729	0.466		
*P. subsulphureus*	−2.237	0.151	−14.790	<0.001		
**(b)**						
**Museum body size (PC1: 68.7%)**					0.66	0.84
Intercept	−0.405	0.069	−5.806	<0.001		
Absolute latitude	0.108	0.006	15.911	<0.001	0.18	
Elevation	0.594	0.043	13.527	<0.001	0.11	
Taxon:					0.23	
*P. chrysoconus + P. pusillus*	−0.023	0.101	−0.232	0.817		
*P. subsulphureus*	−2090	0.147	−14.141	<0.001		
**(c)**						
**Field beak length**					0.26	0.95
Intercept	13.248	0.655	19.917	<0.001		
Absolute latitude	−0.018	0.009	−1.934	0.053	0.01	
Elevation	−0.129	0.067	−1.921	0.054	<0.001	
Field body size (PC1: 63.66%)	0.269	0.046	5.741	<0.001	0.02	
Taxon:					0.20	
*P. chrysoconus + P. pusillus*	−2.632	0.152	−17.214	<0.001		
*P. subsulphureus*	0.269	0.046	5.741	<0.001		
**(d)**						
**Museum beak length**					0.18	0.40
Intercept	11.972	0.049	243.71	<0.001		
Absolute latitude	−0.001	0.005	−0.18	0.853	<0.001	
Elevation	−0.013	0.036	−0.38	0.702	<0.001	
Museum body size (PC1: 68.7%)	0.276	0.031	8.91	<0.001	0.10	
Taxon:					0.04	
*P. chrysoconus + P. pusillus*	−0.281	0.071	−3.92	<0.001		
*P. subsulphureus*	0.419	0.121	3.45	<0.001		

There was less support from variation in appendage size for Allen's rule (Figure 3d–f,j–l) with beak length from field collected data marginally nonsignificantly related to both absolute latitude and elevation (Table 1c). We caution, however, that by including observer as a random factor for consistency with other models violated diagnostic tests, and there was a weakly significant negative association of beak length with elevation in the best fit model (*p* = 0.049), which included observer as a fixed factor, and met model assumptions (Table S4), with longer beaks relative to body size at lower latitudes. *P. chrysoconus* and *P. pusillus* combined had significantly smaller and *P. subsulphureus* a significantly larger beak than *P. bilineatus* relative to body size. The museum collected data also showed a nonsignificant decrease with both absolute latitude and elevation, and specimens of *P. chrysoconus* and *P. pusillus* (pooled together) had significantly shorter, and *P. subsulphureus* a significantly longer beak relative to body size than *P. bilineatus* (Table 1d). Finally, in both datasets, larger body size significantly corresponded to longer beaks.

As predicted, both absolute latitude and elevation have a significant negative effect on annual mean temperature (Table 2a), and consistent with Bergmann's rule predictions, temperature had a significant negative effect on body size, with larger birds found where temperatures are lower (Table 2b). However, there was no support for Allen's rule, with temperature having no effect on beak length after controlling for body size (Table 2c).

**TABLE 2:** Model outputs showing the effects of (a) absolute latitude and elevation on mean annual temperature, (b) temperature and taxon on field body size PC1, (c) temperature, taxon and body size on beak length and (d) latitude, vegetation density (EVI), canopy density (LAI) and elevation on peak song frequency (log‐transformed) of the three tinkerbird taxa combined. The marginal *R* ^2^ and the conditional *R* ^2^ (Cond. *R* ^2^) are also shown

	Estimate	SE	*z*	*p*	Marginal *R* ^2^	Cond. *R* ^2^
**(a)**						
**Annual temperature**					0.36	0.95
Intercept	25.198	0.332	75.86	<0.001		
Absolute latitude	−0.144	0.023	−6.09	<0.001	0.12	
Elevation	−1.808	0.150	−12.02	<0.001	0.28	
**(b)**						
**Field body size (PC1: 63.66%)**					0.34	0.84
Intercept	−0.206	0.185	−1.110	0.267		
Annual temperature	−0.490	0.073	−6.659	<0.001	0.14	
Taxon:					0.20	
*P. chrysoconus + P. pusillus*	0.042	0.138	0.306	0.760		
*P. subsulphureus*	−2.447	0.155	−15.709	<0.001		
**(c)**						
**Field beak length**					0.26	0.95
Intercept	13.075	0.646	20.226	<0.001		
Annual temperature	0.052	0.057	0.923	0.356	<0.001	
Taxon:					0.25	
*P. chrysoconus + P. pusillus*	−2.739	0.143	−19.133	<0.001		
*P. subsulphureus*	0.271	0.198	1.365	0.172		
Field body size (PC1: 63.66%)	0.227	0.040	5.652	<0.001	0.007	
**(d) Log10‐peak frequency**						
**All Taxa**					0.78	0.96
Intercept	6.990	0.004	1426.5	<0.001		
Absolute latitude	−0.002	0.0006	−3.2	0.001	<0.001	
Elevation	−0.021	0.002	−9.7	<0.001	0.03	
EVI	−0.0007	0.002	−0.3	0.738	<0.001	
Taxon:					0.25	
*P. chrysoconus + P. pusillus*	0.211	0.006	32.8	<0.001		
*P. subsulphureus*	0.246	0.007	33.2	<0.001		
*P. chrysoconus + P. pusillus* × absolute latitude	−0.001	0.0006	−1.6	0.109		
*P. subsulphureus* × absolute latitude	0.004	0.001	2.3	0.021		
*P. chrysoconus + P. pusillus* × EVI	−0.010	0.003	−3.3	0.001		
*P. subsulphureus* × EVI	−0.002	0.003	−0.7	0.490		

### Geographical variation in tinkerbird song

**FIGURE 4:**
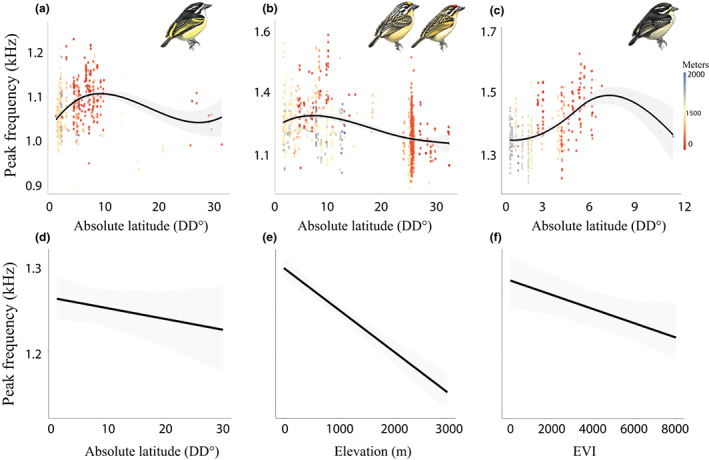
Variation in peak frequency along latitudinal and elevational gradients in (a) *P. bilineatus*, (b) *P. chrysoconus* and *P. pusillus* combined and (c) *P. subsulphureus*. Elevation is represented by the colour scale legend. The lower panels show model effects of (d) absolute latitude, (e) elevation and (f) EVI on peak frequency for the four species modelled together. Grey shaded area indicates 95% confidence interval. Tinkerbird illustrations courtesy of Lynx Edicions (del Hoyo et al., 2020)

Tinkerbirds sang lower pitched songs at both higher latitudes and elevation consistent with a Bergmann's rule effect on body size influencing song frequency. Also, both *P. chrysoconus* and *P. pusillus* combined and *P. subsulphureus* sang higher frequency songs than *P. bilineatus* (Table 2d, Figure 4). There was a significant positive interaction of *P. subsulphureus* with latitude, on peak frequency, indicating the effects of latitude on its song are significantly offset from its effects on *P. bilineatus* song. There was also a significant negative interaction of *P. chrysoconus* and *P. pusillus* with EVI on peak frequency, demonstrating they sing lower frequency songs compared to *P. bilineatus* as vegetation density increases. There were no significant effects of VCF or LAI on song pitch, except for a positive interaction of *P. subsulphureus* song frequency with LAI in the full song model for all‐species combined (Table S2e) but this interaction was not included in the best‐supported model.

Models per species corroborated that both *P. bilineatus*, and *P. chrysoconus* with *P. pusillus* combined sang higher frequency songs at lower latitudes, while elevation was negatively correlated with peak frequency in all taxa (Table S5). The effect of latitude was strongest in *P. chrysoconus* and *P. pusillus* (partial *R* ^2^ = 0.20, ~83% of the marginal *R* ^2^ = 0.24 attributed to predictors), again reflected by a striking pattern of increasing frequency from South Africa northwards towards the equator followed by decreasing frequency towards the northernmost populations in the Horn of Africa (Figure S1). Taken together, our models show that tinkerbirds sing higher pitched songs at lower elevation (*P. bilineatus* partial *R* ^2^ = 0.13, ~92% of variance explained by fixed factors [14%]), *P. subsulphureus* (partial *R* ^2^ = 0.21, 67% of variance explained [31%]) and *P. chrysoconus* and *P. pusillus* (partial *R* ^2^ = 0.05, ~20% of marginal *R* ^2^ [0.24]), and except for *P. subsulphureus*, at lower latitudes. Furthermore, vegetation structure affected song in *P. chrysoconus* and *P. pusillus* only, with EVI negatively associated with peak frequency (partial *R* ^2^ = 0.03, ~12% of a marginal *R* ^2^ of 0.24), but with a lower effect size than for ecogeographical predictors.

### Phylogenetic signal

We detected weak and nonsignificant phylogenetic signals in song frequency and morphology from both field collected data and museum morphometrics (Table S6).

## DISCUSSION

Body size varies across a latitudinal gradient as predicted by Bergmann's rule, and this may underpin continental‐scale song frequency variation in tinkerbirds. Consistent with the strong negative relationship between body size and peak frequency found within several taxa (Kirschel, Zanti, Harlow, et al., 2020; Mikula et al., 2021; Uribarri et al., 2020), tinkerbird song pitch is lower at higher latitudes and elevation, where birds are on average larger, than at lower latitudes and elevation. Furthermore, vegetation density had a significant negative effect on tinkerbird song frequency in some models, providing support for AAH in shaping acoustic signals in birds with innate songs.

The power of the association between Bergmann's rule and song frequency is especially evident when examining the relative contribution of latitude and elevation in model variance at the taxon level. Latitude played a major role in song frequency variation of *P. chrysoconus* and *P. pusillus* and elevation had the strongest effect on both *P. bilineatus* and *P. subsulphureus* song. By contrast, latitude had a significant positive interaction effect on *P. subsulphureus* song frequency in the all‐species model, but not in its taxon‐specific model, suggesting this positive relationship was significant only in comparison with the effect of latitude on *P. bilineatus* song. This result in *P. subsulphureus* is likely a consequence of its smaller latitudinal range (Kirschel, Nwankwo, Seal, & Grether, 2020), strictly within Afrotropical rainforest across the Equator, with latitudinal patterns following Bergmann's rule not expected within the tropics (Huston & Wolverton, 2011). The latitudinal range was especially restricted in our song data for this taxon (all within 6.83° to −1.00° except for three recordings from Angola) and results might have differed had we analysed recordings from further sources covering a wider range. Nevertheless, elevation had significant effects on song pitch for all three taxa, and the overall effects attributable to Bergmann's rule were an order of magnitude greater than those attributable to vegetation density and the AAH. The latter effects were only supported by a negative relationship of EVI, representing greenness and canopy structure, with *P. chrysoconus* and *P. pusillus* song frequency.

We also found a pattern of relatively smaller tinkerbird beaks at higher latitudes and higher elevation, though this was weakly significant in the best‐fit field‐based model (Table S4) and nonsignificant in the museum‐based model. In other words, tinkerbirds tend to have longer beaks towards the Equator and at lower elevation, after correcting for body size. This provides limited evidence for variation in beak size according to Allen's rule but is consistent with a global pattern of higher relative beak length in the tropics across all birds (Tobias et al., 2022). Nowicki (1987) proposed that the suprasyringeal vocal tract in birds has resonating properties: the longer the tract, which includes the buccal cavity, the lower its resonating frequency. However, song frequency patterns did not reflect Allen's rule predictions, with peak frequency higher towards the Equator and at lower elevation, where the vocal tract is relatively longer. Predictions according to Bergmann's rule thus have a stronger effect than those of Allen's rule on song pitch. Furthermore, tinkerbirds appear not to open their beaks when they sing (ANGK, MS, SL *personal observation*), instead likely projecting their tonal pulses by producing airflow in an expiratory direction through the syrinx from inflated air sacs, in a mechanism similar to other birds that lack vocal learning, such as doves (Suthers, 2004). Beak length may thus not play any role in resonating frequencies in such birds. Instead, peak frequency is likely more intimately related with air sac pressure variation, musculature and hence body size (Beckers et al., 2003).

Taken together, these results provide compelling evidence of a primary role of latitude and elevation in shaping song frequency in tinkerbirds, which we suggest is a by‐product of body size variation predicted by Bergmann's rule. Bird song plays a crucial role in bird ecology, functioning in mate attraction, territory defence and synchronisation of breeding behaviour (Bradbury & Vehrencamp, 2011; Catchpole & Slater, 1995). Frequency is a key feature of bird song. Given that it is negatively correlated with body size, it could be sexually selected if individuals with lower‐pitched songs are perceived as more competitive (Mikula et al., 2021). Several studies have supported this assumption, showing that individuals with lower‐pitched songs dominate intrasexual territorial contests, are more attractive to females, and have higher fitness (Brumm & Goymann, 2017; Hardouin et al., 2007; Kirschel, Zanti, Harlow, et al., 2020). Therefore, any mechanisms that lead to consistent variation in frequency might shape interactions among and within species over time, with potential evolutionary consequences.

Body size is just one of the mechanisms proposed to contribute to the evolution of song pitch in birds. Another is the interaction among related species, and in *P. bilineatus* and *P. subsulphureus* it has driven character displacement facilitating species recognition in populations where the species coexist (Kirschel, Blumstein, & Smith, 2009). Although those species' songs diverge where they coexist, the pattern emerges after controlling for the effects of their relative distributions either side of an elevational gradient, but at a continental scale, the climatic effects predicted by Bergmann's rule outweigh any local community effects. Nevertheless, further work would be needed to tease apart relative influences of ecological factors, including relative densities of interacting species at the local community level. This would require intensive work relating the effects of local temperature and locally sampled habitat structure variables with morphometrics of birds whose songs have been subsequently recorded, thus facilitating study of direct causal links from environment to morphology and song. It was not possible here because most songs in our dataset could not be associated with biometrics of ringed individuals (and certainly not with museum skins), a key requirement of methods such as path analysis that could assess causal links.

At regional and local scales, song pitch has been widely hypothesised to adapt to the acoustic properties of the environment (Wiley & Richards, 1978, 1982). Although the hypothesis has been supported across a range of taxa, including birds (Ey & Fischer, 2009), a recent global study across 5000 passerine species has brought this into question (Mikula et al., 2021). We also found limited support for AAH, with an association of EVI with peak frequency in *P. chrysoconus* and *P. pusillus*, suggesting some adaptation of song pitch to habitat structure over a continental scale in these species with a wider ecological niche, from acacia savanna to dense forest, across the continent. Given the innate nature of tinkerbird song (Lukhele et al., 2022), this result might suggest stronger environmental pressure on vocalisation of birds with limited vocal flexibility, as proposed by other studies on birds that lack vocal learning (Bertelli & Tubaro, 2002; McCracken & Sheldon, 1997). Mikula et al. (2021) focused on passerines, the vast majority of which develop songs through cultural learning, and that ability may obscure acoustic adaptation. A targeted study comparing song learners with non‐learners over different spatial scales is certainly warranted to test for the relative effects of AAH on their songs.

Although songs are innate in tinkerbirds, and compared to vocal learners, greater similarity is expected among songs of closely related species, we detected only a weak non‐significant phylogenetic signal in peak frequency, meaning that sister species might not sing at more similar frequencies. There are several possible explanations for this. First, relatedness might have a stronger effect on temporal patterning than pitch. Indeed, songs of sister taxa *P. pusillus* and *P. chrysoconus* involve production of a single note repeated continuously at a constant tempo, while sister taxa *P. subsulphureus* and *P. bilineatus* both arrange their songs temporally in bouts of notes (see Figure 2d). Second, just like there might be differences between individual gene trees and species trees (Edwards et al., 2007), genetically determined songs might depend on a subset of genes whose gene trees might not correspond with the species tree, leading to discordance between songs and phylogenetic trees, as found in *P. bilineatus* by Nwankwo et al. (2018). Third, sister taxa may not necessarily be most similar to one another in body size in a genus, especially when closely related species occur in sympatry and undergo morphological character displacement (Grant & Grant, 2006; Kirschel, Blumstein, & Smith, 2009). That is the case here with *P. subsulphureus* smallest and *P. bilineatus* largest of the taxa included in this study. Instead, we suggest climate drives selection on body size, which is what is then implicated in driving continent‐wide patterns in song pitch. Yet, with a larger number of species, within‐clade species are typically morphologically more similar than among clades (Pigot et al., 2020), but in our study interspecific variation and number of species might simply be too low to detect a phylogenetic signal.

Predictable patterns in behavioural traits based on biogeographical and ecological predictors can help us predict how such traits may respond to increasing environmental change (Trisos et al., 2020). Bergmann's rule defines how endothermic animals may evolve because of climatic differences primarily in temperature. The AAH posits that frequencies are expected to increase in more open habitats, such as where deforestation reduces vegetation density. Coupling the effects of AAH with those of temperature, we expect global warming to further increase song pitch in these birds in predictable ways. Because tinkerbird songs are innate, continent‐wide patterns of variation in their song pitch may serve as a baseline for future studies on the effects of environmental change on the communication signals of birds and other endotherms around the world.

## AUTHORSHIP

MS and AK designed the study. All authors performed fieldwork and AK measured all museum specimens. MS performed spatial, acoustic and statistical analysis under the guidance of AK. MS wrote the first draft of the manuscript with support from AK. All authors revised the manuscript and substantially contributed to the final draft.

### PEER REVIEW

The peer review history for this article is available at https://publons.com/publon/10.1111/ele.14102.

## Supporting information


Appendix S1

## Data Availability

Data and R code used for analysis are available on Figshare online repository (https://doi.org/10.6084/m9.figshare.20066225).
