# Supplementary material for: Continent‐wide patterns of song variation predicted by classical rules of biogeography
Source: Ecol Lett. 2022 Sep 20;25(11):2448–62. doi: 10.1111/ele.14102 (PMC9826498; doi:10.1111/ele.14102)
Supplement: Supplementary file 1 — Appendix S1 [file ELE-25-2448-s001.docx]

SUPPLEMENTAL MATERIAL

**Continent-wide patterns of song variation predicted by classical rules of biogeography**

Matteo Sebastianelli^1^, Sifiso M. Lukhele^1^, Emmanuel C. Nwankwo^1^, Louis Hajioannou^1^ and Alexander N. G. Kirschel^1,2,3^

^1^Department of Biological Sciences, University of Cyprus, PO Box 20537, Nicosia 1678, Cyprus

^2^University of California Los Angeles, Department of Ecology and Evolutionary Biology, Los Angeles, CA, USA

^3^Edward Grey Institute, Department of Zoology, University of Oxford, South Parks Road, Oxford OX1 3PS, UK.

**Table of contents:**

1. **Supplementary methods**
   1. **Quantifying observer bias**
   2. **Acoustic data and song analysis**
   3. **Environmental remote sensing data and interpolation**
   4. **Model selection process**

**2. Supplementary tables (1-5)**

**3. Supplementary figures**

**4. Supplementary references**

**1. SUPPLEMENTAL METHODS**

- 1. **Quantifying observer bias**

To evaluate the extent to which variability of measurements taken by different observers affected our model estimates we followed a two-step approach. We first fitted a null gaussian GLMM on each individual measurement with species, subspecies and individual bird ID as nested random effects and then another null Gaussian GLMM on the same variables but this time with observer as random factor. We then calculated R^2^ values using the MuMin package in R (Barton 2019) as in (Sheard *et al.* 2020) and (Pigot *et al.* 2020). R^2^ values showed that interspecific and individual differences explained 84% of the variation in tarsus, 88% in wing, 99% in tail and 79% in body mass; whereas the observer random effect accounted for 6% of the variation in tarsus length, 24% in wing chord, 30% in tail and 14% in body mass.

As a second step, we visually inspected measurements that exceeded three standard deviations (± 3 SD) the mean of each species (Tobias *et al.* 2022). We then retained the outlier values that were considered arising from natural variation, and removed those caused by obvious observer errors (e.g. decimal misplacement).

- 1. **Acoustic data and song analysis**

Field recordings were obtained using a variety of equipment, including Sennheiser microphones (ME67, ME88, MKH 8020 with Telinga parabola, MKH 8050 or MKH 8070) attached to a Marantz PMD 670 or 661 digital solid-state recorder or Sony TCD5M cassette recorder and these were then saved as 16-bit, 44.1 or 48 kHz WAV files. For recordings of *P. bilineatus* and *P. subsulphureus* which were measured previously in studies focusing on those species (Kirschel *et al.* 2009b; Nwankwo *et al.* 2018; Sebastianelli *et al.* 2021), peak frequency was calculated from several song bouts per individual using power spectra for each 1-3 s song bout, produced using a Fast Fourier transformation of 4096 with a 10.8 Hz frequency resolution in Raven Pro v.1.3 – 1.6 (Center for Conservation Bioacoustics 2019). The recordings attributed to *P. chrysoconus* and *P. pusillus* based on forecrown plumage, as well as those from other sources for *P. bilineatus* and *P. subsulphureus,* were imported into Raven Pro 1.6 and individual notes detected using the built-in band limited automated energy detector (BLED), which detects sounds when the amplitude in a specified band of frequency exceeds a specific signal to noise ratio of background noise (in dB). Note detections were visually inspected for false positives and modified where necessary to match the actual begin and end time of each note. During this process, we used the waveform view as a reference to avoid potential error associated with visual interpretation of the spectrogram as well as to annotate notes that were missed by the BLED. For these measurements, as per Sebastianelli et al. (2021), we set Discrete Fourier Transform (DFT size 65,536) to obtain a high-frequency resolution for spectral measurements, and used Hann windows and 50% overlap. We then extracted peak frequency from each detection. Although we used different approaches to measure tinkerbird songs, differences in frequency resolution (e.g. different spectrogram settings between more recent and historical measurements) are negligible and unlikely to affect model inference.

- 1. **Environmental remote sensing data and interpolation**

As mentioned in the main text, we used several variables representing habitat structure to assess the potential effect of niche specialisation and acoustic adaptation to different habitats. From the Moderate-Resolution Imaging Spectroradiometer (MODIS), we obtained raster files for enhanced vegetation index (EVI) at 250 m^2^ resolution (MODIS/Terra Vegetation Indices 16-Day L3 Global 250m Grid V061), leaf area index (LAI, 500 m^2^ resolution) (MODIS/Terra Leaf Area Index/FPAR 8-Day L4 Global 500m Grid V061) and percent tree cover (VCF, 250 m^2^ resolution) (MODIS/Terra Vegetation Continuous Fields Yearly L3 Global 250m Grid V006). LAI is an indicator of canopy density, EVI is a measure of vegetation greenness and canopy structure as well as incorporating LAI (Fernández-Martínez *et al.* 2015), but has also been used as an indicator of primary productivity. VCF represent the percentage of tree cover. Elevation data were obtained at 30 m^2^ resolution (1-arc second) from the global Shuttle Radar Topography Mission (SRTM) (Farr *et al.* 2007), whereas annual mean temperature (0.5 degrees latitude 𝗑 0.5 degrees longitude resolution) was extracted from GHCN-CAMS Gridded 2m Temperature (Fan & van den Dool 2008).

For point localities with spatial uncertainty (those from museum collections and online / private recording repositories), to avoid overspecificity of extracted values to identified coordinates, we interpolated values in ARCGIS, which calculates a value for a specific cell including those from adjacent cells with valid values using bilinear interpolation.

- 1. **Model selection process**

We selected our best fit models via backward selection from full models by selecting different combinations of fixed and random factors until the lowest corrected Akaike Information Criterion score was obtained (Mazerolle 2019). This process helps minimise collinearity among predictors. We caution that in the global model for song peak frequency we detected high VIF in the interaction term between absolute latitude and taxon in the best fit model, Such high VIF are probably a consequence of the interaction structure in the model. However, we overcame the collinearity issue by running peak frequency models on each taxon separately (see Table S5 below).

All continuous response variables were standardised to aid model comparison (Schielzeth 2010) using the *scale* function and collinearity among latitude and the remaining covariates checked (Zuur *et al.* 2010) (see Table S2 for full models and variance inflation factors). Model assumptions were also checked and fit confirmed using the *DHARMa* (Harting 2019) and *performance* (Lüdecke *et al.* 2021) packages in R. It is through this process that we identified when using beak length as response variable, we found that models with observer as random factor violated model assumptions. However, model assumptions were met when using observer as fixed factor rather than random. It was our initial intention to run the models for beak length with observer as random factor for consistency with the other body size models. We therefore provide the best model (with observer as fixed factor) in Table 2, but also provide the model with observer as random effect in Table S4.

Visualisation of raw data was affected by the interaction between latitude and elevation with many lower latitude localities at higher elevation, thus obscuring the trends predicted in Fig.1. Therefore, together with raw data (which have been visualised with a *loess* smoothing function in the *ggplot* R package (Wickham & Winston 2016) in the rightmost panel of Fig. 3 and upper panels of Fig. 4 to highlight the combined effect of latitude and elevation), we visualised model effects using the *effects* package (Fox & Weisberg 2018) to illustrate the effect of single covariates separately. Furthermore, to identify the relative effects of each predictor, we computed the proportion of variance explained by fixed factors and estimated the relative contribution of each predictor to the total variance of the best fit models using *partR2* in R (Stoffel *et al.* 2021). It is important to note that this function also partitions the *R*^2^ for combinations of predictors, and therefore the *R*^2^ of the single predictors alone do not sum up to total variance explained by the model.

**2. SUPPLEMENTAL TABLES**

**Table S1.** Summary of the tinkerbird species measured from each ornithological collection. See also detailed material uploaded in the online repository for additional information regarding the museum specimens (museum specimens ID: https://doi.org/10.5061/dryad.905qfttn2).

| **Collection** |  | ***P. bilineatus*** | ***P. chrysoconus*** | ***P. pusillus*** | ***P. subsulphureus*** |
| --- | --- | --- | --- | --- | --- |
| AMNH |  | 3 | 24 | 26 | 0 |
| LACM |  | 131 | 15 | 20 | 35 |
| LSUMNS |  | 10 | 12 | 9 | 0 |
| NMNH |  | 5 | 15 | 21 | 0 |
| YPM |  | 0 | 13 | 25 | 0 |
| UCLA |  | 16 | 0 | 2 | 0 |
| FMNH |  | 112 | 42 | 7 | 0 |
| NHMUK |  | 52 | 17 | 7 | 49 |
| AMNH: American Museum of Natural History; LACM: Natural History Museum of Los Angeles County; LSUMNS: Louisiana State University Museum of Natural Science, NMNH: National Museum of Natural History; YPB: Yale Peabody Museum of Natural History; UCLA: Donald R. Dickey bird collection at UCLA; FMNH: Chicago Field Museum of Natural History; NHMUK: Natural History Museum of the United Kingdom. | | | | | |

**Table S2.** Full models (GLMMs) for field body size PC1 (which includes tarsus, tail, wing length and body mass), and relative beak length with (**a** and **c**) latitude and elevation, and (**b** and **d**) temperature, respectively, and full model (**e**) for peak song frequency. Variance inflation factors (VIF) are reported in the rightmost column.

|  | **Estimate** | **Std. Error** | ***z*** | ***p*** | **VIF** |
| --- | --- | --- | --- | --- | --- |
| **a)** | |  |  |  |  |
| **Field body size (PC1: 63.66%)** | |  |  |  |  |
| Intercept | -1.486 | 0.153 | -9.657 | <0.001 |  |
| Absolute latitude | 0.112 | 0.007 | 14.279 | <0.001 | 1.16 |
| Elevation | 0.373 | 0.066 | 5.611 | <0.001 | 1.17 |
| EVI | 0.009 | 0.032 | 0.280 | 0.780 | 1.03 |
| LAI | 0.034 | 0.045 | 0.747 | 0.455 | 1.15 |
| VCF | 0.069 | 0.047 | 1.452 | 0.147 | 1.19 |
| Taxon: |  |  |  |  | 1.34 |
| *P. chrysoconus + P. pusillus* | -0.035 | 0.129 | -0.276 | 0.783 |  |
| *P. subsulphureus* | -2.293 | 0.153 | -14.894 | <0.001 |  |
|  |  |  |  |  |  |
| **b)** | |  |  |  |  |
| **Field body size (PC1: 63.66%)** | |  |  |  |  |
| Intercept | -0.083 | 0.203 | -0.409 | 0.682 |  |
| Annual temperature | -0.501 | 0.087 | -5.738 | <0.001 | 1.01 |
| EVI | 0.014 | 0.032 | 0.440 | 0.66- | 1.01 |
| LAI | 0.001 | 0.049 | 0.026 | 0.979 | 1.06 |
| VCF | 0.029 | 0.052 | 0.566 | 0.572 | 1.11 |
| Taxon: |  |  |  |  | 1.09 |
| *P. chrysoconus + P. pusillus* | 0.044 | 0.142 | 0.315 | 0.753 |  |
| *P. subsulphureus* | -2.499 | 0.161 | -15.467 | <0.001 |  |
|  |  |  |  |  |  |
| **c)** | |  |  |  |  |
| **Beak length** | |  |  |  |  |
| Intercept | 12.117 | 0.153 | 79.0 | <0.001 |  |
| Absolute latitude | -0.016 | 0.009 | -1.83 | 0.067 | 2.53 |
| Elevation | -0.125 | 0.066 | -1.88 | 0.059 | 1.57 |
| EVI | 0.009 | 0.040 | 0.23 | 0.816 | 1.10 |
| LAI | 0.074 | 0.052 | 1.42 | 0.156 | 1.35 |
| VCF | -0.039 | 0.054 | 1.42 | 0.156 | 1.33 |
| Field body size (PC1: 63.66%) | 0.267 | 0.046 | 5.73 | <0.001 | 2.46 |
| Taxon: |  |  |  |  | 2.83 |
| *P. chrysoconus + P. pusillus* | -2.618 | 0.155 | -16.81 | <0.001 |  |
| *P. subsulphureus* | 0.293 | 0.203 | 1.44 | 0.149 |  |
| Observers: |  |  |  |  | 2.14 |
| Observer 2 | 3.674 | 0.188 | 19.51 | <0.001 |  |
| Observer 3 | -0.420 | 0.157 | -0,030303 | 0.007 |  |
| Observer 4 | 1.303 | 0.153 | 8.48 | <0.001 |  |
| Observer 5 | 0.965 | 0.143 | 6.71 | <0.001 |  |
|  |  |  |  |  |  |
| **d)** | |  |  |  |  |
| **Beak length** | |  |  |  |  |
| Intercept | 11.966 | 0.135 | 88.06 | <0.001 |  |
| Annual temperature | 0.044 | 0.069 | 0.65 | 0.517 | 1.34 |
| EVI | -0.003 | 0.041 | -0.08 | 0.937 | 1.07 |
| LAI | 0.065 | 0.056 | 1.15 | 0.249 | 1.27 |
| VCF | -0.063 | 0.059 | -1.07 | 0.283 | 1.25 |
| Field body size (PC1: 63.66%) | 0.194 | 0.046 | 4.21 | <0.001 | 1.91 |
| Taxon: |  |  |  |  | 2.39 |
| *P. chrysoconus + P. pusillus* | -2.649 | 0.160 | -16.46 | <0.001 |  |
| *P. subsulphureus* | 0.236 | 0.224 | 1.06 | 0.291 |  |
| Observers: |  |  |  |  | 1.81 |
| Observer 2 | 3.890 | 0.204 | 19.00 | <0.001 |  |
| Observer 3 | -0.412 | 0.167 | -2.46 | 0.014 |  |
| Observer 4 | 1.260 | 0.165 | 7.61 | <0.001 |  |
| Observer 5 | 0.977 | 0.150 | 6.51 | <0.001 |  |
|  |  |  |  |  |  |
| **e)** | |  |  |  |  |
| **Log_10_-peak frequency** | |  |  |  |  |
| Intercept | 6.990 | 0.005 | 1369.5 | <0.001 |  |
| Absolute latitude | -0.001 | 0.0006 | -2.9 | 0.004 | 1.30 |
| Elevation | -0.018 | 0.003 | -5.3 | <0.001 | 1.25 |
| EVI | -0.0004 | 0.002 | -0.2 | 0.843 | 1.16 |
| LAI | -0.003 | 0.003 | -1.1 | 0.256 | 1.35 |
| VCF | 0.0009 | 0.003 | 0.3 | 0.801 | 1.50 |
| Taxon: |  |  |  |  | 1.65 |
| *P. chrysoconus + P. pusillus* | 0.207 | 0.007 | 28.4 | <0.001 |  |
| *P. subsulphureus* | 0.243 | 0.011 | 22.1 | <0.001 |  |
| *(P. chrysoconus + P. pusillus)* 𝗑 absolute latitude | -0.001 | 0.0007 | -1.6 | 0.100 |  |
| *P. subsulphureus* 𝗑 absolute latitude | 0.005 | 0.002 | 2.0 | 0.049 |  |
| *(P. chrysoconus + P. pusillus)* 𝗑 elevation | -0.002 | 0.004 | -0.6 | 0.568 |  |
| *P. subsulphureus* 𝗑 elevation | -0.006 | 0.007 | -1.0 | 0.316 |  |
| *(P. chrysoconus + P. pusillus)* 𝗑 EVI | -0.008 | 0.003 | -2.6 | 0.008 |  |
| *P. subsulphureus* 𝗑 EVI | -0.002 | 0.004 | -0.7 | 0.485 |  |
| *(P. chrysoconus + P. pusillus)* 𝗑 VCF | -0.003 | 0.005 | -0.7 | 0.489 |  |
| *P. subsulphureus* 𝗑 VCF | -0.005 | 0.006 | -0.9 | 0.375 |  |
| *(P. chrysoconus + P. pusillus)* 𝗑 LAI | -0.0004 | 0.004 | -0.1 | 0.921 |  |
| *P. subsulphureus* 𝗑 LAI | 0.011 | 0.004 | 2.5 | 0.012 |  |

**Table S3.** Factor loadings of principal components on the three *Pogoniulus* taxa from A) field (Bartlett's test of sphericity; χ^2^ = 1349.31, *p* = <0.001) and B) museum collected measurements (Bartlett's test of sphericity; χ^2^ = 799.08, *p* = <0.001). Keiser-Meyer-Olkin (KMO) index is also reported in the rightmost column.

| **A)** | **PC1** | **PC2** | **PC3** | **PC4** | **KMO** |
| --- | --- | --- | --- | --- | --- |
| **Eigenvalue** | 1.59 | 0.99 | 0.54 | 0.40 |  |
| **% Variance** | 63.66 | 24.65 | 0,07 | 0,04 |  |
| **Mass** | 0.55 | 0.13 | -0,82 | -0.05 | 0.77 |
| **Wing** | 0,56 | -0,24 | 0.29 | 0.72 | 0.67 |
| **Tarsus** | 0.21 | 0.93 | 0.29 | 0.02 | 0.50 |
| **Tail** | 0.56 | -0.23 | 0.39 | -0.68 | 0.69 |
| **B)** | **PC1** | **PC2** | **PC3** |  |  |
| **Eigenvalue** | 1.43 | 0.86 | 0.44 |  |  |
| **% Variance** | 68.7 | 24.8 | 6.5 |  |  |
| **Wing** | 0.62 | -0.38 | -0.68 |  | 0.56 |
| **Tarsus** | 0.44 | 0.89 | -0.09 |  | 0.81 |
| **Tail** | 0.64 | -0.24 | 0.72 |  | 0.55 |

**Table S4.** Best fit GLMM showing the effects of absolute latitude, taxon and elevation on beak length, which includes observer as a fixed factor. The relative importance of the predictors (i.e. marginal R^2^) and the conditional R^2^ (Cond. R^2^) are also reported.

| \|  \| **Estimate** \| **Std. Error** \| ***z*** \| ***p*** \| **Marginal *R*^2^** \| **Cond. *R*^2^** \| \| --- \| --- \| --- \| --- \| --- \| --- \| --- \| \| **Field beak length** \| \|  \|  \|  \| 0.63 \| 0.91 \| \| Intercept \| 12.147 \| 0.151 \| 80.03 \| <0.001 \|  \|  \| \| Absolute latitude \| -0.018 \| 0.009 \| -1.96 \| 0.049 \| <0.001 \|  \| \| Elevation \| -0.128 \| 0.066 \| -1.92 \| 0.054 \| 0.01 \|  \| \| Field body size (PC1: 63.66%) \| 0.269 \| 0.046 \| 5.79 \| <0.001 \| 0.03 \|  \| \| Taxon: \|  \|  \|  \|  \| 0.23 \|  \| \| *P. chrysoconus + P. pusillus* \| -2.647 \| 0.152 \| -17.37 \| <0.001 \|  \|  \| \| *P. subsulphureus* \| 0.293 \| 0.198 \| 1.47 \| 0.140 \|  \|  \| \| Observers: \|  \|  \|  \|  \| 0.33 \|  \| \| Observer 2 \| 3.724 \| 0.182 \| 20.41 \| <0.001 \|  \|  \| \| Observer 3 \| -0.447 \| 0.157 \| -2.84 \| 0.004 \|  \|  \| \| Observer 4 \| 1,322 \| 0.153 \| 8.63 \| <0.001 \|  \|  \| \| Observer 5 \| 1,009 \| 0.138 \| 7.29 \| <0.001 \|  \|  \| |
| --- | --- | --- | --- | --- | --- | --- | --- | --- | --- | --- | --- | --- | --- | --- | --- | --- | --- | --- | --- | --- | --- | --- | --- | --- | --- | --- | --- | --- | --- | --- | --- | --- | --- | --- | --- | --- | --- | --- | --- | --- | --- | --- | --- | --- | --- | --- | --- | --- | --- | --- | --- | --- | --- | --- | --- | --- | --- | --- | --- | --- | --- | --- | --- | --- | --- | --- | --- | --- | --- | --- | --- | --- | --- | --- | --- | --- | --- | --- | --- | --- | --- | --- | --- | --- | --- | --- | --- | --- | --- | --- | --- | --- | --- | --- | --- | --- | --- | --- |

**Table S5.** Best fit GLMM output on the effect of latitude, vegetation density (EVI), canopy density (LAI) and elevation on song peak frequency (log-transformed) of (a) *P. bilineatus*, (b) *P. chrysoconus* and *P. pusillus* combined and c) *P. subsulphureus*. The marginal *R*^2^ and conditional *R*^2^ (Cond. *R*^2^) are also shown.

|  | **Estimate** | **Std. Error** | ***z*** | ***p*** | **Marginal *R*^2^** | **Cond. *R*^2^** |
| --- | --- | --- | --- | --- | --- | --- |
| **a) *P. bilineatus*** | |  |  |  | 0.14 | 0.85 |
| Intercept | 6.987 | 0.005 | 1307.9 | <0.001 |  |  |
| Absolute latitude | -0.001 | 0.0006 | -3.1 | 0.002 | 0.01 |  |
| Elevation | -0.020 | 0.003 | -5.6 | <0.001 | 0.13 |  |
| **b) *P. chrysoconus + P. pusillus*** | | |  |  | 0.24 | 0.85 |
| Intercept | 7.203 | 0.005 | 1379.9 | <0.001 |  |  |
| Absolute latitude | -0.003 | 0.0003 | -9.8 | <0.001 | 0.20 |  |
| Elevation | -0.022 | 0.002 | -7.4 | <0.001 | 0.05 |  |
| EVI | -0.007 | 0.002 | -3.7 | <0.001 | 0.03 |  |
| LAI | -0.002 | 0.001 | -1.6 | 0.111 | 0.001 |  |
| **c) *P. subsulphureus*** | |  |  |  | 0.31 | 0.97 |
| Intercept | 7.224 | 0.010 | 707.3 | <0.001 |  |  |
| Absolute latitude | 0.004 | 0.002 | 1.8 | 0.075 | 0.06 |  |
| Elevation | -0.025 | 0.005 | -4.4 | <0.001 | 0.21 |  |
| EVI | -0.002 | 0.004 | -0.7 | 0.508 | 0.01 |  |

**Table S6.** Table showing results of Blomberg’s K test for phylogenetic signal.

| **Field dataset** | ***K*** | ***z*** | ***p-value*** |
| --- | --- | --- | --- |
| Peak frequency | 0.32 | 1.30 | 0.742 |
| Beak length | 1.43 | 1.273 | 0.259 |
| Mass | 0.46 | 0.35 | 0.742 |
| Tarsus length | 0.50 | 0.198 | 0.411 |
| Tail length | 0.85 | -0.670 | 0.273 |
| Wing length | 1.02 | -0.95 | 0.232 |
| **Museum dataset** | ***K*** | ***z*** | ***p-value*** |
| Beak length | 0.61 | -0.20 | 0.583 |
| Wing length | 0.70 | -0.43 | 0.584 |
| Tail length | 0.32 | 1.33 | 0.742 |
| Tarsus length | 0.32 | 1.33 | 0.742 |

**3. SUPPLEMENTARY FIGURES**

**Figure S1.** Plot with raw data showing latitudinal variation (a) in peak song frequency of *P. chrysoconus* and *P. pusillus* only, and body size of all the species (PC1) from (b) field and (c) museum measurements, respectively.

**4. SUPPLEMENTARY REFERENCES**

Barton, K. (2019). MuMIn: Multi-Model Inference. *Retrieved March*.

Center for Conservation Bioacoustics. (2019). Raven Pro: Interactive Sound Analysis Software (Version 1.6) [Computer software].

Fan, Y. & van den Dool, H. (2008). A global monthly land surface air temperature analysis for 1948-present. *Journal of Geophysical Research Atmospheres*, 113.

Farr, T.G., Rosen, P.A., Coro, E., Crippen, R., Duren, R., Hensley, S., *et al.* (2007). The shuttle radar topography mission. *Reiviews of geophysics*, 45.

Fernández-Martínez, M., Garbulsky, M., Peñuelas, J., Peguero, G. & Espelta, J.M. (2015). Temporal trends in the enhanced vegetation index and spring weather predict seed production in Mediterranean oaks. *Plant Ecology*, 216, 1061–1072.

Fox, J. & Weisberg, S. (2018). Visualizing Fit and Lack of Fit in Complex Regression Models with Predictor Effect Plots and Partial Residuals. *Journal of Statistical Software*, 87, 1–27.

Harting, F. (2019). DHARMa: Residual Diagnostics for Hierarchical (Multi-Level / Mixed) Regression Models. R package version 0.4.5. https://CRAN.R-project.org/package=DHARMa.

Lüdecke, D., Ben-Shachar, M.S., Patil, I., Waggoner, P. & Makowski, D. (2021). Performance: assessment of regression models performance. *The Journal of Open Source Software*, 6, 3139.

Mazerolle, M.J. (2019). AICmodavg: Model selection and multimodel inference based on (Q)AIC(c).

Pigot, A.L., Sheard, C., Miller, E.T., Bregman, T.P., Freeman, B.G., Roll, U., *et al.* (2020). Macroevolutionary convergence connects morphological form to ecological function in birds. *Nature Ecology & Evolution*, 4, 230–239.

Schielzeth, H. (2010). Simple means to improve the interpretability of regression coefficients. *Methods in Ecology and Evolution*, 1, 103–113.

Sebastianelli, M., Blumstein, D.T. & Kirschel, A.N.G. (2021). Higher-pitched bird song towards the coast supports a role for selection in ocean noise avoidance. *Bioacoustics*, 31, 41–58.

Sheard, C., Neate-Clegg, M.H.C., Alioravainen, N., Jones, S.E.I., Vincent, C., MacGregor, H.E.A., *et al.* (2020). Ecological drivers of global gradients in avian dispersal inferred from wing morphology. *Nature Communications*, 11.

Stoffel, M.A., Nakagawa, S. & Schielzeth, H. (2021). partR2: Partitioning R2 in generalized linear mixed models. *PeerJ*, 9.

Tobias, J.A., Sheard, C., Pigot, A.L., Devenish, A.J.M., Yang, J., Neate-Clegg, M.H.C., *et al.* (2022). AVONET: morphological, ecological and geographical data for all birds. *Ecology Letters*, 1–17.

Wickham, H. & Winston, C. (2016). “Package ‘ggplot2’.” Create elegant data visualisations using the grammar of graphics.

Zuur, A.F., Ieno, E.N. & Elphick, C.S. (2010). A protocol for data exploration to avoid common statistical problems. *Methods in Ecology and Evolution*, 1, 3–14.
